# Interactive Effects of Long-term Exposure to Air Pollutants on SARS-CoV-2 Infection and Severity: A Northern Italian Population-based Cohort Study

**DOI:** 10.1097/EDE.0000000000001792

**Published:** 2024-09-24

**Authors:** Giovanni Veronesi, Sara De Matteis, Camillo Silibello, Emanuele M. Giusti, Walter Ageno, Marco M. Ferrario

**Affiliations:** a Department of Medicine and Surgery, Research Center in Epidemiology and Preventive Medicine, University of Insubria, Varese, Italy; b Department of Health Sciences, University of Milan, Milan, Italy; c Arianet, Milano, Italy.

**Keywords:** Air pollution, SARS-CoV-2, Cohort, Italy, Interactive effect, Long-term exposure, Urban

## Abstract

**Background:**

We examined interactions, to our knowledge not yet explored, between long-term exposures to particulate matter (PM_10_) with nitrogen dioxide (NO_2_) and ozone (O_3_) on severe acute respiratory syndrome coronavirus 2 (SARS-CoV-2) infectivity and severity.

**Methods:**

We followed 709,864 adult residents of Varese Province from 1 February 2020 until the first positive test, COVID-19 hospitalization, or death, up to 31 December 2020. We estimated residential annual means of PM_10_, NO_2_, and O_3_ in 2019 from chemical transport and random-forest models. We estimated the interactive effects of pollutants with urbanicity on SARS-CoV-2 infectivity, hospitalization, and mortality endpoints using Cox regression models adjusted for socio-demographic factors and comorbidities, and additional cases due to interactions using Poisson models.

**Results:**

In total 41,065 individuals were infected, 5203 were hospitalized and 1543 died from COVID-19 during follow-up. Mean PM_10_ was 1.6 times higher and NO_2_ 2.6 times higher than WHO limits, with wide gradients between urban and nonurban areas. PM_10_ and NO_2_ were positively associated with SARS-CoV-2 infectivity and mortality, and PM_10_ with hospitalizations in urban areas. Interaction analyses estimated that the effect of PM_10_ (per 3.5 µg/m^3^) on infectivity was strongest in urban areas [hazard ratio (HR) = 1.12; 95% CI =1.09, 1.16], corresponding to 854 additional cases per 100,000 person-years, and in areas at high NO_2_ co-exposure (HR = 1.15; 1.08, 1.22). At higher levels of PM_10_ co-exposure, the protective association of O_3_ reversed (HR =1.32, 1.17, 1.49), yielding 278 additional cases per µg/m^3^ increase in O_3_. We estimated similar interactive effects for severity endpoints.

**Conclusions:**

We estimate that interactive effects between pollutants exacerbated the burden of the SARS-CoV-2 pandemic in urban areas.

In 2023, the detrimental effects of long-term exposure to outdoor airborne pollutants on health were on the agenda at the Conference of the Parties 28 agenda for the first time.^1^ Latest estimates for Europe indicate that 94% of the population live at airborne particulate matter (PM) concentrations above the 2021 WHO guideline levels,^2^ resulting in 117,000 premature deaths.^3^ Over the years, an extensive body of research has suggested that there is a causal link between air pollution and morbidity from chronic, noncommunicable diseases.^4,5^

Less established is the link between long-term exposure to PM_2.5_ and PM_10_ (e.g., particulate matter with aerodynamic diameter less than 2.5 and 10 µm, respectively), nitrogen dioxide (NO_2_), and ozone (O_3_) with infectious diseases, including the severe acute respiratory syndrome coronavirus 2 (SARS-CoV-2) threats. A number of mechanisms support the biologic plausibility of the association, including the well-established role of airborne pollutants in prolonged inflammation, downregulation of the immune system, and over-expression of angiotensin-converting enzyme-2 receptors in the lungs with impairment of alveoli macrophages to regulate the inflammation response.^6–8^

Nonetheless, prospective epidemiologic studies with individual-level data have been so far insufficient to fully elucidate the relationships of air pollution with SARS-CoV-2 infectivity and severity.^9^ One nationwide study in Denmark^10^ on a population at very low exposure levels showed a consistent, increased risk of infection, hospitalization, and mortality for increased exposure to PM_2.5_ and NO_2_; in contrast, the associations for O_3_ were protective, remaining negative also in bi-pollutant models. In the same pandemic period but at higher exposures, two distinct UK-biobank studies^11,12^ and one in the northern Italian city of Varese^13^ found positive associations between PMs and NO_2_ for infectivity only, while two studies in Catalonia^14,15^ and one in the city of Rome^16^ confirmed positive associations for severity endpoints only. Some of these studies also reported negative associations of O_3_ with SARS-CoV-2 endpoints^13,15^ in single-pollutant analyses, the estimates becoming positive after controlling for PM^13^ and NO_2_.^15^

The heterogeneity in estimates at different exposure levels, and in single-city versus country-wide studies, may suggest the presence of interactive effects of air pollutants with SARS-CoV-2 infectivity and severity. Synergistic interactions between acute exposure to PM_2.5_ and O_3_ with total, respiratory and cardiovascular mortality were recently reported by Liu and colleagues^17^ in pooled analyses of 372 cities, while interactive effects of long-term exposures on the SARS-CoV-2 pandemic have not been formally investigated so far.

In the present prospective cohort study with individual-level data, we expand to the entire Varese Province—comprising of both urban and rural areas—our previous analysis limited to the city of Varese,^13^ and we further add COVID-19 hospitalizations and mortality to SARS-CoV-2 infection as endpoints. In particular, we aim to investigate the presence of interactive effects of long-term exposures to PM_10_, NO_2_, and O_3_ with the study endpoints in urban and nonurban areas, with different levels of exposure.

## METHODS

### Study Population and Study Period

The target population comprised adult (≥18 years old) residents both on 1 January 2019 and on 31 December 2019 in the Province of Varese (Lombardia region, northern Italy) registered with the Regional Health Service (n = 725,443). The province of Varese covers 1200 square kilometers, comprising 138 municipalities, most of which are medium to small in size. Individuals’ residential addresses as of 31 December 2019 were geocoded. Geocoding quality was high, with only 1.7% of individuals allocated to the centroid of the residential municipality. We accessed regional healthcare databases to collect information on COVID-19, hospital discharge records, and outpatient drug prescriptions through a unique, anonymized identification code. The study period preceded the opening of the vaccination season in the region, as well as the circulation of the first variant of concern (alpha),^18^ to avoid such sources of heterogeneity in study endpoints. We excluded n = 5562 individuals living in residential nursing homes and n = 10,017 individuals without active regional assistance as of 1 February 2020 (start date of follow-up), leaving a final sample of 709,864 individuals.

### Environmental Exposure

Study exposures are the 2019 annual mean values of PM_10_, NO_2_, and O_3_ estimated at a spatial resolution of 1 km^2^ from the combined use of chemical-transport and random-forest models, a methodology already used in the context of large epidemiologic studies.^19,20^ The choice of a prepandemic year was consistent with the literature,^10–16^ to avoid modifications in pollutants’ levels due to lockdown and to mobility restriction periods occurring in 2020. Briefly, for each pollutant the chemical-transport model combined data from emission inventories and meteorologic conditions to produce concentration fields that were further integrated with observed ground levels from monitoring stations using data fusion techniques.^19^ Then, random-forest algorithms downscaled the “data fused” fields at the spatial resolution of 1 km^2^ using spatial-temporal predictors such as population density, traffic, land use, and surface greenness.^20^ Because of the scanty availability of data monitoring stations in the study area resulting in unexpected weak correlations with the other pollutants, we excluded PM_2.5_ from the present analyses. Full details, including some metrics of models’ performance, are reported in Supplementary eAppendix 1; http://links.lww.com/EDE/C181. We then assigned individual exposures based on the grid cell centroid closest to the subject’s geocoded address.

### Endpoints

The Regional Health Authority collected information on COVID-19 from several sources, including public and private hospitals, local health agencies, and accredited laboratories.^13^ In Lombardia Region, both symptomatic patients and asymptomatic individuals identified through contact tracing were tested. We defined the infectivity endpoint as the occurrence of the first positive nasopharyngeal swab specimens tested with real-time reverse-transcriptase–polymerase-chain-reaction assays targeting different genes (E, RdRp, and M) of SARS-CoV-2 before 31 December 2020. In agreement with the literature,^10,15^ we defined the COVID-19 hospitalization endpoint as a hospital admission (including admission to the emergency departments) for any reason within 28 days from the detection of the first positive swab or within 48 hours before the swab to account for minor discrepancies in dates. Finally, COVID-19 mortality was defined as a death for any cause within 30 days of the first positive swab.^10,15^

### Demographic, Socio-economic, and Clinical Characteristics

Age and gender were available in the regional healthcare databases. We used an area-based deprivation index developed and validated in Italy for epidemiologic studies,^21^ available at census section level. The index uses individual data of the latest general population and housing census of 2011, and it considers five dimensions of social and material deprivation: low education, unemployment, renting, household crowding, and living in a single-parent family. We categorized the index into fifths using region-specific quintiles. As a measure of population density, we used the Eurostat^22^ classification of the degree of urbanization (“cities,” “towns and suburbs,” “rural areas”), available at a municipality level. We defined medications (one prescription during the year 2019) and history of comorbidities (at least one hospital discharge between 2015 and 2019) for each individual from relevant regional healthcare databases, as in our previous research,^13^ specific codes being reported in the eAppendix 1; http://links.lww.com/EDE/C181.

### Statistical Analysis

Starting 1 February 2020, for each of the three endpoints the subjects contributed to follow-up until the date of the event, or censoring due to death for any cause, residency change outside the Varese Province, or 31 December 2020, whichever came first. We estimated the separate effect of each pollutant on the endpoints from single-pollutant Cox regression models. Potential confounders and mediators from literature^6–8^ were identified using directed acyclic graphs, which we report in eFigure 1; http://links.lww.com/EDE/C181 an example for the infectivity endpoint. We estimated the associations between the potential confounders with the exposure estimated using linear and the endpoints using Cox regression models. According to these, we adjusted the analyses for age, sex, degree of urbanization, deprivation index, selected comorbidities, and active treatments. We accounted for nonlinear effects of age on the infectivity using a polynomial of degree 3 and the mortality endpoints using a polynomial of degree 2. Due to the low number of individuals and events in rural areas, we dichotomized the degree of urbanization as urban and nonurban, the latter comprising towns, suburbs, and rural areas. The hazard ratios (HR) are reported for one interquartile range width increase in the exposures.

To assess the presence of interactive effects, we adopted a stratified approach^17,23^ consisting of regression models with one pollutant, one stratification variable indicating co-exposure, and their interaction terms. We used Cox models to formally test for interactions on the relative scale, and Poisson models (SAS Proc NLMIXED; https://support.sas.com/kb/37/344.html) to further estimate the additional number of events due to interaction at the sample mean values for pollutants and covariates. Since our previous study investigated one single city,^13^ we first tested the homogeneity of air pollutant effects on the endpoints in urban and nonurban areas. Urbanicity represents not only different pollutant exposures but also factors potentially related to the pandemic such as population density. To allow comparison with previous studies,^10,15^ we also report the HRs for the first (from March to May 2020) and the second (June–December 2020) pandemic waves in urban and nonurban areas, from time-stratified Cox models. In sensitivity analyses of the interactive effects by urbanization, we report the results from bi-pollutant Cox regression models.

Then, to better characterize the nature of the interactions overall, we looked at the association of each pollutant by levels of the other, classified according to the lowest (≤25%), middle (26–74%), and highest (≥75%) quartile. In these analyses, we did not consider NO_2_ and O_3_ together since nitrogen oxides are in the formation pathway of O_3_.^24,25^ We formally tested the homogeneity of effect by strata in Cox regression models with interaction terms between the pollutant and the co-pollutant levels (2 df Wald chi-square test), again using Poisson modeling for interaction on the additive scale. Furthermore, since interactive effects so far have been investigated in urban settings only,^17^ we assessed associations of each pollutant and study endpoints by urbanization-specific strata of co-pollutant exposures (in quintiles). For these analyses, the HRs are reported for a 1 µg/m^3^ increase in pollutants, as quintile intervals most of the time were narrower than the overall interquartile range. We conducted the statistical analyses using SAS (9.4 release) and R (version 4.1.2) for the figures.

## RESULTS

We observed 41,065 cases of first SARS-CoV-2 infection, corresponding to a rate of 6424 per 100,000 person-years. Of these, 78% were symptomatic and 22% were identified through screening/contact tracing; while 97% of cases were confirmed through molecular and 3% through antigenic swabs. N = 5203 cases (rate: 747 per 100,000 py) were hospitalized, 59% of which required admission in the intensive care unit (ICU) or mechanical ventilation (n = 3061, rate: 439 per 100,000 py; eTable 1 in eAppendix 1; http://links.lww.com/EDE/C181); and 1543 (rate: 220 per 100,000 py) died. The completeness of case-ascertainment in our sample compared with official counts of cases (source: https://github.com/CSSEGISandData/COVID-19) is presented as eFigure 2 in the eAppendix 1; http://links.lww.com/EDE/C181. The independent associations between demographic and clinical characteristics of the study sample with the endpoints are reported in eTable 2; http://links.lww.com/EDE/C181. Infectivity risk decreased with increased age, and it was lower in men than in women. Conversely, hospitalization and mortality risks increased with age and were higher in men than in women. Higher deprivation was generally associated with increased risk of all the endpoints. Urbanization was associated with SARS-CoV-2 infection, the risk being lower in towns and rural areas than in cities; but not with COVID-19 hospitalization or mortality. Use of treatments and presence of comorbidities were consistently associated with increased risks of infectivity and COVID-19 severity endpoints, except for the lipid-lowering treatments variable, which was no longer considered in the analyses.

### Air Pollutants Distributions

Over the year 2019, the annual mean ± SD level of PM_10_ was 24.3 ± 3.0 µg/m^3^, NO_2_ was 26.1 ± 5.4 µg/m^3^, and O_3_ was 52.1 ± 5.8 µg/m^3^ (eTable 3 in eAppendix 1; http://links.lww.com/EDE/C181). Notably, in the entire province the exposure ranges were 13.4–28.2 µg/m^3^ for PM_10_ and 8.3–37.0 µg/m^3^ for NO_2_, indicating a wide variation in exposures. Air pollutant distributions had a clear gradient according to the degree of urbanization (Table 1). In contrast to the rural areas mainly located in the north, the southern part of the province is constituted by a cluster of closely situated cities, collectively forming a densely populated conurbation, with exceptionally high annual mean levels of PM_10_ (26.2 µg/m^3^) and NO_2_ (30.5 µg/m^3^), and low levels of O_3_ (48.2 µg/m^3^; eTable 3 and eFigures 3–5 in eAppendix 1; http://links.lww.com/EDE/C181). PM_10_ and NO_2_ were positively correlated with each other (Spearman correlation coefficient: 0.84) and negatively with O_3_ (−0.87 and −0.89, respectively; eTable 3; http://links.lww.com/EDE/C181). We observed no consistent gradient in air pollutant distributions by deprivation index, nor by demographic and clinical characteristics of the individuals (Table 1).

**TABLE 1.** Association Between Demographic and Clinical Characteristics of the Study Sample with Annual Mean Values of Air Pollutant (Year 2019)

	PM_10_ [µg/m^3^]	NO_2_ [µg/m^3^]	O_3_ [µg/m^3^]
	beta^a^ (95% CI)	beta^a^ (95% CI)	beta^a^ (95% CI)
Age, years	−0.045 (−0.018, −0.011)^b^	0.002 (−0.004, 0.007)^b^	0.03 (0.02, 0.03)^b^
Men vs. women	0.019 (0.008, 0.029)	−0.006 (−0.023, 0.011)	−0.03 (−0.05, −0.01)
Degree of urbanization			
Cities	REF	REF	REF
Towns and suburbs	−3.46 (−3.48, −3.45)	−7.45 (−7.47, −7.43)	7.14 (7.12, 7.16)
Rural	−8.54 (−8.58, −8.50)	−14.47 (−14.53, −14.42)	15.87 (15.78, 15.96)
Deprivation index—quintiles (regional)			
1—Least deprived	REF	REF	REF
2	−0.09 (−0.11, −0.08)	−0.10 (−0.13, −0.08)	−0.52 (−0.55, −0.49)
3	−0.23 (−0.25, −0.21)	0.31 (0.29, 0.34)	−0.81 (−0.84, −0.78)
4	−0.68 (−0.70, −0.66)	0.10 (0.07, 0.13)	−0.42 (−0.45, −0.38)
5—Most deprived	−0.62 (−0.64, −0.60)	0.11 (0.08, 0.14)	0.18 (0.15, 0.22)
History of drug treatment, yes vs. no^c^			
Diabetes	0.01 (−0.01, 0.04)	−0.02 (−0.06, 0.02)	−0.07 (−0.11, −0.02)
Antihypertensive	0.02 (0.01, 0.04)	0.0002 (−0.024, 0.024)	−0.07 (−0.10, −0.04)
Lipid-lowering	−0.02 (−0.04, 0.004)	−0.07 (−0.10, −0.04)	0.02 (−0.014, 0.05)
Treatment for obstructive airway diseases	0.05 (0.03, 0.06)	0.02 (−0.008, 0.05)	−0.04 (−0.07, 0.003)
Positive history of, yes vs. no^d^			
Coronary heart disease	0.021 (−0.02, 0.06)	0.06 (−0.01, 0.12)	−0.03 (−0.11, 0.05)
Stroke	−0.08 (−0.13, −0.04)	−0.10 (−0.17, −0.02)	0.20 (0.12, 0.29)
Cancer	0.023 (−0.009, 0.05)	0.07 (0.02, 0.12)	−0.09 (−0.15, −0.03)
Chronic obstructive pulmonary disease	−0.20 (−0.26, −0.13)	−0.26 (−0.36, −0.16)	0.58 (0.46, 0.70)

a Change in air pollutant annual mean, from linear regression models.

b Beta for 10-year increase in age.

c At least one prescription during the year 2019. Anatomical Therapeutic Chemical classification (ATC) classes: diabetes (A10); antihypertensive (C02, C03, C07, C08, and C09); lipid-lowering (C10); treatment for obstructive airway diseases (R03).

d At least one hospital discharge record between 1 Jan 2015 and 31 Dec 2019. ICD-IX codes: coronary heart disease (410–414); stroke (430–438); cancer (140–208); COPD (490–496).

### Interactive Effects by Urbanization

The HRs [with 95% confidence intervals (CIs)] for the association of air pollutants with the study endpoints, in the entire province, and by urban versus nonurban areas are reported in Table 2. In the entire province, after the adjustment for covariates, PM_10_ and NO_2_ were associated with an increased risk of SARS-CoV-2 infection and COVID-19 mortality. The associations with COVID-19 hospitalization, and with hospitalizations requiring ICU or mechanical ventilation, were also generally positive for PM_10_ and NO_2._ Conversely, increased O_3_ exposure was associated with decreasing risks for all the endpoints. These associations were confirmed in sensitivity analyses excluding individuals with exposure attributed at the centroid of their municipality of residency (1.7%; eTable 4; http://links.lww.com/EDE/C181).

**TABLE 2.** Association Between Long-term Exposure to Air Pollutants and the Study Endpoints, in the Entire Province and in Urban and Nonurban Areas

	Province^a^			Urban^b^			Non-urban^b^			Urban^*^pollutant interaction p-value^b^
	HR	95%CI		HR	95%CI		HR	95%CI		
Infectivity										
PM_10_	1.02	1.01	1.03	1.12	1.09	1.16	0.99	0.98	1.01	<0.001
NO_2_	1.02	1.00	1.05	1.03	1.00	1.07	1.02	0.99	1.05	0.48
O_3_	0.95	0.93	0.97	0.92	0.89	0.95	0.97	0.94	1.00	0.01
Hospitalization										
PM_10_	1.00	0.96	1.04	1.18	1.08	1.29	0.96	0.91	1.00	<0.001
NO_2_	1.02	0.95	1.08	1.06	0.96	1.17	0.99	0.91	1.07	0.28
O_3_	0.96	0.90	1.02	0.91	0.83	1.00	1.00	0.92	1.08	0.12
Of these: hospitalization requiring ICU/mechanical ventilation										
PM_10_	0.99	0.94	1.05	1.13	1.01	1.27	0.96	0.90	1.02	0.01
NO_2_	1.05	0.97	1.14	1.10	0.97	1.25	1.02	0.92	1.14	0.38
O_3_	0.94	0.87	1.01	0.85	0.75	0.96	1.01	0.91	1.11	0.04
Mortality										
PM_10_	1.09	1.01	1.17	1.22	1.04	1.42	1.05	0.96	1.14	0.11
NO_2_	1.18	1.05	1.32	1.18	0.99	1.41	1.17	1.00	1.37	0.97
O_3_	0.91	0.82	1.02	0.89	0.76	1.05	0.93	0.80	1.08	0.74

a Single-pollutant Cox regression models, adjusting for age, sex, urbanization (urban vs. nonurban), deprivation index (quintiles), positive history of coronary heart disease, stroke, cancer, chronic obstructive pulmonary disease, treatment for diabetes, antihypertensive treatment, treatment for obstructive airway diseases. For the infectivity and the mortality endpoints, age was modeled as a polynomial of degree 3 and 2, respectively.

b Single-pollutant Cox regression models, adjusting for age, sex, urbanization (urban vs. nonurban), deprivation index (quintiles), positive history of coronary heart disease, stroke, cancer, chronic obstructive pulmonary disease, treatment for diabetes, antihypertensive treatment, treatment for obstructive airway diseases + air pollutant

* urbanization interaction term (1 df). For the infectivity and the mortality endpoints, age was modeled as a polynomial of degree 3 and 2, respectively.

HR: Hazard ratios for 1 interquartile range width (IQRw) increase in province-wide air pollutants. IQRw values: PM_10_=3.5 µg/m^3^; NO_2_=8.7 µg/m^3^; O_3_=9.7 µg/m^3^

We observed spatial heterogeneity in the effect of PM_10_ on SARS-CoV-2 infection and hospitalization (urban area*pollutant interaction test *P* value <0.001), the associations being stronger in urban rather than nonurban areas (Table 2). For the infectivity endpoint, the HR was 1.12 (95% CI = 1.09, 1.16) in urban areas and 0.99 (95% CI = 0.98, 1.01) in nonurban areas. On the additive scale, in urban areas we estimated 854 (95% CI = 600, 1107) additional SARS-CoV-2 cases per 100,000 person-years due to the interaction; these cases correspond to 13% of the event rate. Similarly, we estimated 174 (95% CI = 86, 261) additional COVID-19 hospitalizations (23% of the event rate), and 74 (95% CI = 13, 134) additional COVID-19 hospitalizations requiring ICU/mechanical ventilation (17% of the event rate). Finally, we also estimated a higher protective effect of O_3_ in urban areas. The associations for PM_10_, NO_2_, and O_3_ with the study endpoints were generally consistent across pandemic waves, although slightly attenuated during the second wave in urban areas (eTable 5; http://links.lww.com/EDE/C181). Results from sensitivity analyses using bi-pollutant models (eTables 6–8; http://links.lww.com/EDE/C181) support the independent roles of PM_10_ in urban settings and NO_2_ in nonurban areas. In addition, the associations between O_3_ and the study endpoints reversed when adjusting for PM_10_ in urban areas.

### Interactive Effects by Co-pollutant Levels

The stratified associations of PM_10_, NO_2_, and O_3_ with the study endpoints by levels of co-pollutant are reported in Table 3. The associations of PM_10_ with SARS-CoV-2 infectivity increased in higher quartiles of NO_2_ (interaction test *P* value < 0.0001; the HR in the highest quartile being 1.15, 95% CI = 1.08, 1.22); and in lower quartiles of O_3_ (*P* value < 0.0001, the HR in the lowest quartile being 1.15, 95% CI = 1.06, 1.25). Conversely, the effect of NO_2_ was the strongest at low levels of PM_10_ (HR = 1.09; 95% CI = 1.03, 1.16; interaction test *P* value <0.0001). Finally, in the highest fourth of PM exposure, the effect of O_3_ on SARS-CoV-2 on infectivity became positive and statistically significant (HR = 1.32; 95% CI = 1.17, 1.49; interaction test *P* < 0.0001). On the additive scale, we estimated 278 (95% CI = 184, 372) additional cases per 100,000 py per each µg/m^3^ increase in O_3_ due to the interaction.

**TABLE 3.** Hazard Ratios for SARS-CoV-2 Infectivity, Hospitalization and Mortality Associated with One Interquartile Range Increase in PM_10_, NO_2_ and O_3_, by Levels of Co-pollutants

	PM_10_ by NO_2_ levels	NO_2_ by PM_10_ levels	PM_10_ by O_3_ levels	O_3_ by PM_10_ levels
	HR (95% CI)	HR (95% CI)	HR (95% CI)	HR (95% CI)
Infectivity				
≤25th pct	0.96 (0.94, 0.99)	1.09 (1.03, 1.16)	1.15 (1.06, 1.25)	0.88 (0.83, 0.93)
26–75th pct	1.09 (1.05, 1.13)	1.06 (1.01, 1.10)	1.07 (1.04, 1.11)	0.92 (0.88, 0.95)
>75th pct	1.15 (1.08, 1.22)	0.87 (0.82, 0.93)	0.96 (0.94, 0.99)	1.32 (1.17, 1.49)
Heterogeneity *P* value^a^	<0.0001	<0.0001	<0.0001	<0.0001
Hospitalization				
≤25th pct	0.92 (0.85, 0.99)	1.00 (0.85, 1.18)	1.09 (0.85, 1.38)	1.06 (0.91, 1.23)
26–75th pct	1.11 (1.02, 1.21)	1.10 (0.98, 1.24)	1.17 (1.08, 1.27)	0.87 (0.79, 0.97)
>75th pct	1.28 (1.07, 1.53)	0.81 (0.68, 0.98)	0.88 (0.81, 0.95)	1.85 (1.32, 2.58)
Heterogeneity *P* value^a^	0.0002	0.02	<0.0001	<0.0001
Mortality				
≤25th pct	0.92 (0.79, 1.07)	0.95 (0.69, 1.31)	1.27 (0.82, 1.97)	1.32 (1.00, 1.74)
26–75th pct	1.21 (1.04, 1.41)	1.25 (1.01, 1.54)	1.31 (1.12, 1.52)	0.93 (0.78, 1.12)
>75th pct	1.10 (0.82, 1.48)	0.88 (0.64, 1.22)	0.99 (0.86, 1.15)	1.43 (0.78, 2.61)
Heterogeneity *P* value^a^	0.04	0.10	0.03	0.07

HR, hazard ratios for 1 interquartile range width (IQRw) increase in Province-wide air pollutants. IQRw values: PM_10_=3.5 µg/m^3^; NO_2_=8.7 µg/m^3^; O_3_=9.7 µg/m^3^.

Hazard Ratios estimates from Cox models including age, sex, urbanization (urban vs. nonurban), deprivation index (quintiles), positive history of coronary heart disease, stroke, cancer, chronic obstructive pulmonary disease, treatment for diabetes, antihypertensive treatment, treatment for obstructive airway diseases, air pollutant, co-pollutant levels (in three classes) and air pollutant*co-pollutant levels interaction. For the infectivity and the mortality endpoints, age was modeled as a polynomial of degree 3 and 2, respectively.

a 2df Wald chi-square test.

When we further looked at the stratified associations by considering co-pollutant levels by degree of urbanization (Figure), we mostly observed consistent effects in urban and nonurban areas characterized by the same co-pollutant exposure level. The progressive increase of O_3_ HRs with infectivity by quintile levels of PM_10_ is shown in panel D of Figure. The stratified analyses for COVID-19 hospitalizations and mortality confirmed the presence of interactive effects of PM_10_ by levels of NO_2_ and O_3_; and of NO_2_ and O_3_ by levels of PM_10_, but the estimates have lower precision (Table 3 and eFigures 6–7 in eAppendix 1; http://links.lww.com/EDE/C181).

**FIGURE.**
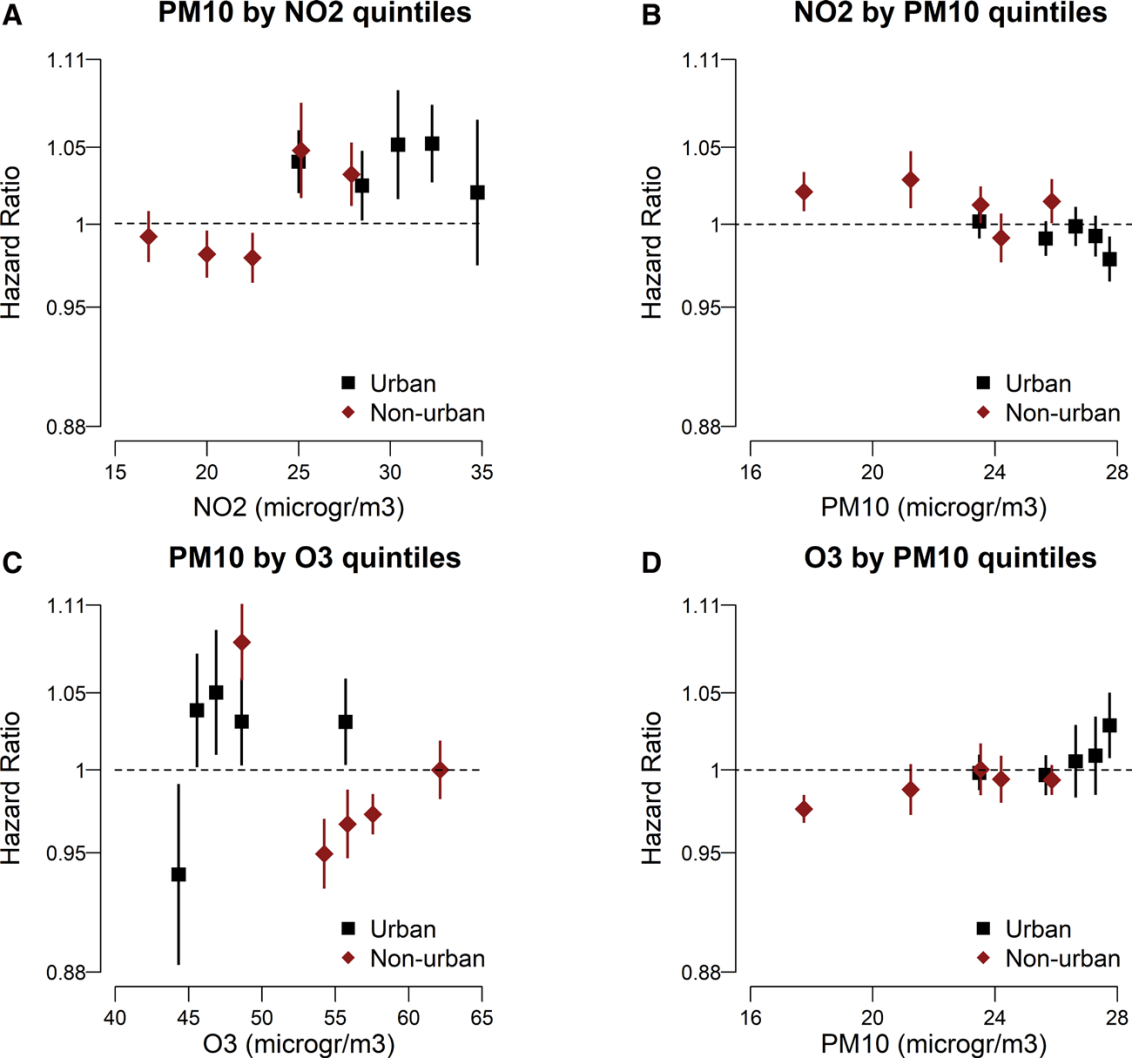
Modeling of SARS-CoV-2 infectivity risks due to 1 µg/m^3^ increase in one pollutant by urban and nonurban specific quintiles of the co-pollutant. A, Effect of PM_10_ by quintiles of NO_2_ co-exposure. B, Effect of NO_2_ by quintiles of PM_10_ co-exposure. C, Effect of PM_10_ by quintiles of O_3_ co-exposure. D, Effect of O_3_ by quintiles of PM_10_ co-exposure.

## DISCUSSION

In this large prospective study of adult residents in the Varese Province, we found associations between increasing PM_10_ and NO_2_ exposure levels with increased risks of SARS-CoV-2 infection and mortality, and between PM_10_ and COVID-19 hospitalizations in urban areas. Associations were the strongest in urban settings, particularly so for PM_10_. Annual mean PM_10_ was 1.6 times and NO_2_ was 2.6 times higher than the latest WHO limits.^2^ Thus, we confirmed at higher exposure levels the associations observed in Denmark,^10^ where about 40% of the population was instead exposed to NO_2_ levels below the limit of 10 µg/m^3^.

### Interactive Effects of PM_10_ with NO_2_ and O_3_

With respect to existing literature,^10–15^ we explored the additional notion of interactive effects of chronic exposure to PM_10_ with NO_2_ and O_3_ on SARS-CoV-2 risk, the former having an increased estimate of detrimental effect when combined with high levels of NO_2_, and with lower levels of O_3_. This mixture occurs in cities, characterized by elevated PM and NOx emissions, the latter mainly represented by nitrogen oxide that reacts with O_3_ (O_3_ titration) leading to NO_2_ formation.^24,26^ The interactions then are consistent with each other, and support both the observed heterogeneity of PM_10_ effects by urbanization and the independent effect of PM_10_ in urban settings as emerging from bi-pollutant models. Notably, in urban areas, the interactive estimated effects of pollutants substantially contributed to the total burden of the pandemic by as much as 13–23% of infectivity and hospitalization rates.

Conversely, the estimated effect of NO_2_ was the highest for lower levels of PM_10_, as in nonurban areas characterized by lower levels of nitrogen oxide and particulate due to reduced levels of traffic and other anthropogenic sources. Since about 65% of the population in Italy lives in nonurban areas,^27^ our finding corroborates the Italian nationwide EpiCovAir ecologic study, which estimated a larger effect of NO_2_ vs. PM on SARS-CoV-2 infectivity.^28^

Furthermore, our interaction analyses cast a new perspective on the role of O_3_ in the SARS-CoV-2 pandemic. In single-pollutant analyses in the literature, O_3_ was associated with a reduced risk of SARS-CoV-2 infectivity^10,13^ and severity,^10,15^ as we observed in our study. The negative associations have been attributed to several reasons, including the photochemical origin of O_3_ that depends on solar radiation, which itself has a virucidal effect^29^; and to the ability of O_3_ to inactivate SARS-CoV-2 in indoor experimental studies.^30^ On the other hand, O_3_ has detrimental health effects on the respiratory tract due to its pro-inflammatory capacity and oxidative activity^31^; pathways shared with PM.^6–8^ In bi-pollutant models, O_3_ remained protective at low levels of PM_10_, as in Denmark^10^; but the association reversed at higher levels of PM_10_, as in the city of Varese^13^ and in urban areas in the present article. Although with some caution due to the different methodological approaches, in line with these observations, the findings we report in Table 3 and panel D of Figure for the infectivity endpoint indicate that O_3_ maintains a protective effect only at low levels of PM. This suggests that in the presence of elevated PM co-exposure, the detrimental effects of O_3_ through inflammatory and oxidative pathways may completely counterbalance and overcome the putative advantages due to the mechanisms of virus inactivation. Instead, in SARS-CoV-2-infected individuals, O_3_ increases the risk of severe disease and of adverse outcomes, in particular death, already at the lowest levels of PM observed in our study (again Table 3 and eFigure 7; http://links.lww.com/EDE/C181). A synergic interaction between PM_2.5_ and O_3_ has been recently reported on other adverse health outcomes, including daily mortality for cardiovascular and respiratory diseases^17^ and preterm birth for co-exposures occurring during the entire pregnancy period.^32^ Liu and colleagues^17^ in particular report small increases in all-cause and cardiovascular disease mortality risks associated with increasing O_3_ already at low levels of PM_2.5_. These findings are supported by experimental studies investigating the biological plausibility of a combined effect of PM and O_3_ through pathways involving inflammation, oxidative stress, macrophage activity, and endothelial dysfunction.^33^ Most of these mechanisms are also invoked for the link between chronic exposure to air pollution and SARS-CoV-2.^6–8^ In addition, sulfuric acid, related to ambient PM, enhances the severity of pulmonary lesions produced by O_3_.^34^ Finally, one study found a peculiar combination of high PM with weather conditions favoring the accumulation of O_3_ beneath the worst pandemic wave in the Tokyo metropolitan area.^35^ Future studies looking at the specific chemical composition of PM^36^ may further elucidate the nature of the interactions we have detected.

### Strengths and Limitations

Study strengths include the design and the availability of a variety of individual-level data on exposure, outcomes, and potential confounders, in a large population residing in urban and rural areas, in one of the most polluted regions in Europe. We were able to investigate COVID-19 severity endpoints on the entire population potentially reducing collider biases.^37^ Study weaknesses include the definition of long-term exposure based on a 1-year average of pollutants, but air quality reports from the Regional Agency for Environmental Protection indicate no substantial temporal trends from 2014 on in the study region. To minimize misclassification of long-term exposure, we defined our study population by individuals who stayed in the study area during the entire exposure year 2019. However, we have no further information on time since residency at the address used to attribute exposure. We were not able to investigate here the effects of PM_2.5_. However, in our previous study in the city of Varese, the effects of PM_2.5_ and PM_10_ on SARS-CoV-2 infectivity were overlapping.^13^ As in many observational studies, our associations require a cautious interpretation in causal terms as they are exposed to residual confounding. We have no information on individual-level socio-economic status, but its effect as a residual confounder on top of multi-dimensional area-based deprivation is not well-established.^10,11,15^ We were not able to consider the potential role of mobility and social interactions on the pandemic dynamics, although mobility restrictions were in force during about half of our study period. Although we considered a variety of potential confounders, we lack data on obesity and lifestyle habits, including smoking. The differential distributions of these factors across exposure levels have not been well documented. Furthermore, our results are adjusted for socioeconomic status and for major noncommunicable chronic diseases having obesity and smoking as causal factors.

## CONCLUSIONS

In conclusion, we estimated interactive effects between PM_10_ and NO_2_, and between O_3_ and PM_10_, on SARS-CoV-2 incidence and on COVID-19 severity. The total burden on disease and the overload of hospitalizations requiring intensive care due to long-term exposure to multiple pollutants may then exceed the sum of single exposures, thus sustaining the inclusion of environmental health among the key factors for public health preparedness to the next pandemic crisis.^38^ The presence of similar interactive effects on other health outcomes should be carefully evaluated, to have more complete estimates of the impact of residential and traffic emissions in cities and highly-conglomerated areas.^39^

## Acknowledgments

This study was carried out according to the Lombardy Region laws on the use of regional healthcare databases for research activities (D.g.r. XI/491, 02/08/2018; Decreto n. 16256, 12/11/2019), and in particular on COVID-19 disease (D.g.r XI/3019, 30/03/2020). We thank the Osservatorio Epidemiologico Regionale and the Azienda Regionale per l’Innovazione e gli Acquisti (ARIA) S.p.a. for supporting this research. We thank Guido Lanzani, Regional Agency for Environmental Protection (ARPA Lombardia), for providing the data on exposures and insights on their interpretation. Finally, we thank Hannah Forest for helping with English language editing.

## Supplementary Material
