# Supplementary material for: Interactive Effects of Long-term Exposure to Air Pollutants on SARS-CoV-2 Infection and Severity: A Northern Italian Population-based Cohort Study
Source: Epidemiology. 2024 Sep 24;36(1):11–9. doi: 10.1097/EDE.0000000000001792 (PMC11594552; doi:10.1097/EDE.0000000000001792)

Interactive effects of long-term exposure to air pollutants on SARS-CoV-2 infection and severity: a northern Italian population-based cohort study

eAppendix 1 - Methods, Tables and Figures

Definition of the environmental exposure

Monthly averaged ground level concentrations for $PM_{2.5}$, PM_{10}, NO_2, and O_3 for the year 2019 were provided by the Regional Agency for Environmental Protection over a domain covering the entire region at a spatial resolution of 4 km^2. These concentration fields have been produced by a modelling system based on the chemical transport model FARM (Flexible Air quality Regional Model(1)), that includes modules aimed at producing meteorological fields and related turbulence parameters, processing data from emission inventories and considering the contribution to pollution levels coming from the surrounding areas (boundary-conditions). Such system also implements data fusion techniques(2) to integrate the concentration fields produced by FARM with the observations collected by the regional air quality network. To downscale the "data-fused" fields to a target spatial resolution of 1 Km^2, a machine learning Random Forest (RF) model has been developed with several spatial-temporal predictors, such as population, land-use, surface greenness and vehicular traffic as input, according to a method developed in the context of large epidemiological studies(3). The monthly averaged concentration fields produced by the RF model were then further aggregated to estimate yearly exposures to be used in the present study. While the overall performance of the RF model on the studied domain was satisfactory (percent of explained variability, R^2, of 0.85, 0.86, 0.77 and 0.93 for $PM_{2.5}$, PM_{10}, NO_2, and O_3), a zoom within the Varese Province showed a weak correlation coefficient between $PM_{2.5}$ and the other pollutants, particularly prominent in its urban areas. This can be directly linked to the limited availability in the area of interest of monitoring stations for this pollutant. This limitation can undermine the spatial representativeness of the network, and led to a reduction in the dataset size used for model training, consequently influencing the assessment and performance evaluation of $PM_{2.5}$. As such, we excluded $PM_{2.5}$ from the present analyses.

Definition of drug treatments and comorbidities: ATC and ICD-IX codes.

Selected drug medications were traced from the Regional healthcare database, with use defined as at least one prescription during the year 2019. We defined the treatment categories using the Anatomical Therapeutic Chemical classification (ATC) code: diabetes (ATC A10); anti-hypertensive (ATC: C02, C03, C07, C08 and C09); lipid-lowering drugs (ATC C10); and drugs for respiratory diseases (including LABA, SABA, inhaled corticosteroids, and other medicaments;

ATC R03). History of comorbidities was defined as at least one hospital discharge record between 2015 and 2019 with the following discharge diagnosis (any field out of the available six, ICD-IX° code): coronary heart disease (CHD; 410.x-414.x); stroke (430.x-438.x); cancer (140.x-208.x); chronic obstructive pulmonary disease (COPD) and allied conditions (490.x-496.x).

References

1. Silibello C, Calori G, Brusasca G, Giudici A, Angelino E, Fossati G, et al. Modelling of PM10 concentrations over Milano urban area using two aerosol modules. Environ Model Softw. 2008 Mar 1;23(3):333–43.

2. Silibello C, Bolignano A, Sozzi R, Gariazzo C. Application of a chemical transport model and optimized data assimilation methods to improve air quality assessment. Air Qual Atmosphere Health. 2014 Sep;7(3):283–96.

3. Gariazzo C, Carlino G, Silibello C, Renzi M, Finardi S, Pepe N, et al. A multi-city air pollution population exposure study: Combined use of chemical-transport and random-Forest models with dynamic population data. Sci Total Environ. 2020 Jul 1;724:138102.

eTable 1: Characteristics of the hospitalization for COVID-19, in urban and in non-urban areas.

	**Entire Province**	**Urban areas**	**Non-urban areas**
No. of SARS-CoV-2 infection	41065	19933	21132
No. of hospitalized cases (% on infected)	5203 (12.7%)	2546 (12.8%)	2657 (12.6%)
Age, mean±SD	68.4±15.9	68.4±16.2	68.4±15.5
Men, n (%)	3117 (59.9%)	1523 (59.8%)	1594 (60.0%)
Hospitalization requiring ICU or mechanic ventilation, n (% on hospitalized cases)	3061 (58.8%)	1486 (58.4%)	1575 (59.3%)
Median length of hospitalization, days [p25, p75]	10 [4, 18]	10 [5, 18]	10 [4, 19]
In-hospital death, n (%)	1307 (22.5%)	635 (24.9%)	672 (25.3%)
History of drug treatment, n (%)[a]			
Diabetes	963 (18.5%)	452 (17.8%)	511 (19.2%)
Anti-hypertensive	3172 (61.0%)	1525 (59.9%)	1647 (62.0%)
Treatment for obstructive airway diseases	825 (15.9%)	400 (15.7%)	425 (16.0%)
Positive history of, n (%)[b]			
Coronary heart disease (CHD)	350 (6.7%)	167 (6.6%)	183 (6.9%)
Stroke	273 (5.3%)	126 (5.0%)	147 (5.5%)
Cancer	404 (7.8%)	214 (8.4%)	190 (7.2%)
Chronic Obstructive Pulmonary Disease *(COPD)*	176 (3.4%)	75 (3.0%)	101 (3.8%)

Abbreviations: SD=Standard Deviation; ICU= Intensive Care Unit.

[a]: At least one prescription during the year 2019. ATC classes: diabetes (A10); anti-hypertensive (C02, C03, C07, C08 and C09); lipid-lowering (C10); treatment for obstructive airway diseases (R03). [b]: at least one hospital discharge record between Jan, 1st 2015 and Dec, 31st 2019. ICD-IX codes: coronary heart disease (410-414); stroke (430-438); cancer (140-208); COPD (490-496).

eTable 2: Association between demographic and clinical characteristics of the study sample with the study endpoints.

	All subjects	Infectivity		Hospitalization		Mortality	
		Value	HR[a] (95%CI)	Value	HR[a] (95%CI)	Value	HR[a] (95%CI)
N	709864	41065	-	5203	-	1543	-
Person-years	639161.0	30452.8	-	3517.6	-	1101.2	-
Age, years	52.8 (18.6)	50.4 (18.0)	0.982 (0.981; 0.983)[d]	68.4 (15.9)	1.043 (1.040;1.045)[e]	80.0 (9.5)	1.109 (1.102; 1.116)[f]
Men, n (%)	341817 (48.2%)	19506 (47.5%)	0.951 (0.933; 0.970)	3117 (59.9%)	1.75 (1.66; 1.85)	969 (62.9%)	2.35 (2.11; 2.61)
Degree of urbanization							
Cities	338108 (47.6%)	19933 (48.5%)	REF	2546 (48.9%)	REF	779 (50.6%)	REF
Towns and suburbs	356693 (50.3%)	20349 (49.6%)	0.96 (0.94; 0.98)	2542 (48.9%)	0.97 (0.92; 1.02)	727 (47.2%)	0.93 (0.84; 1.03)
Rural	15063 (2.1%)	783 (1.9%)	0.87 (0.81; 0.94)	115 (2.2%)	1.04 (0.86; 1.25)	35 (2.3%)	1.08 (0.77; 1.51)
Deprivation index							
1 - Least deprived	141340 (19.9%)	7946 (19.4%)	REF	931 (17.9%)	REF	284 (18.4%)	REF
2	155116 (21.9%)	9148 (22.2%)	1.04 (1.01; 1.07)	1126 (21.6%)	1.13 (1.03; 1.23)	342 (22.2%)	1.15 (0.98; 1.34)
3	153838 (21.7%)	9013 (22.0%)	1.03 (1.00; 1.06)	1139 (21.9%)	1.15 (1.06; 1.26)	325 (21.1%)	1.11 (0.95; 1.30)
4	130702 (18.4%)	7221 (18.8%)	1.04 (1.01; 1.08)	1008 (19.4%)	1.20 (1.10; 1.31)	297 (19.3%)	1.18 (1.01; 1.40)
5 - Most deprived	128868 (18.2%)	7237 (17.6%)	0.98 (0.95; 1.01)	999 (19.2%)	1.21 (1.10; 1.32)	293 (19.0%)	1.19 (1.01; 1.40)
Treatment, n (%)[b]							
Diabetes	41903 (5.9%)	2507 (6.1%)	1.19 (1.14; 1.24)	963 (18.5%)	1.59 (1.48; 1.72)	392 (25.4%)	1.73 (1.54; 1.95)
Anti-hypertensive	203812 (28.7%)	11034 (26.9%)	1.13 (1.10; 1.16)	3172 (61.0%)	1.41 (1.31; 1.51)	1227 (79.6%)	1.48 (1.29; 1.69)
Lipid-lowering	98909 (13.9%)	5364 (13.1%)	1.04 (1.00; 1.08)	1693 (32.5%)	1.06 (0.99; 1.14)	621 (40.3%)	0.97 (0.86; 1.08)
Obstructive airway diseases	61626 (8.7%)	3980 (9.7%)	1.16 (1.13; 1.20)	825 (15.9%)	1.33 (1.19; 1.50)	297 (19.3%)	1.27 (1.11; 1.46)
Positive history of, n (%)[c]							
Coronary heart disease	12938 (1.8%)	818 (2.0%)	1.19 (1.11; 1.28)	350 (6.7%)	1.33 (1.19; 1.50)	161 (10.5%)	1.53 (1.28; 1.82)
Stroke	10002 (1.4%)	601 (1.5%)	1.21 (1.11; 1.31)	273 (5.3%)	1.45 (1.28; 1.64)	136 (8.8%)	1.53 (1.28; 1.83)
Cancer	20325 (2.9%)	1169 (2.9%)	1.18 (1.11; 1.25)	404 (7.8%)	1.46 (1.32; 1.62)	172 (11.2%)	1.52 (1.29; 1.78)
COPD	5246 (0.75%)	359 (0.9%)	1.26 (1.13; 1.40)	176 (3.4%)	1.48 (1.26; 1.74)	94 (6.1%)	1.71 (1.36; 2.15)

[a]: Hazard Ratio, from multivariate Cox regression models. [b]: At least one prescription during the year 2019. ATC classes: diabetes (A10); anti-hypertensive (C02, C03, C07, C08 and C09); lipid-lowering (C10); treatment for obstructive airway diseases (R03). [c]: at least one hospital discharge record between Jan, 1st 2015 and Dec, 31st 2019. ICD-IX codes: coronary heart disease (410-414); stroke (430-438); cancer (140-208); COPD (490-496). [d]: Age as a polynomial of degree 3. HR for 1-year increase in age from 51 to 50
[e]: Age as a linear term. HR for 1-year increase in age [f]: Age as a polynomial of degree 3. HR for 1-year increase in age from 81 to 80

eTable 3: Descriptive statistics and Spearman correlation coefficients for 2019 annual mean of air pollutants (in µg/m^3), in the entire Province and in urban and non-urban areas.

	Mean	SD	Min	Max	Median	P25	P75	IQR	Correlation coefficient (S)		
									PM_{10}	NO_2	O_3
**Entire Province (n=709864)**											
PM_{10} (µg/m^3)	24.3	3.0	13.4	28.2	24.6	23.3	26.7	3.5	1		
NO_2 (µg/m^3)	26.1	5.4	8.3	37.0	26.4	21.8	30.4	8.7	0.835	1	
O_3 (µg/m^3)	52.1	5.8	41.9	79.6	53.2	46.6	56.4	9.7	-0.870	-0.894	1
**Urban areas (n=338108)**											
PM_{10} (µg/m^3)	26.2	1.6	18.3	28.2	26.7	25.4	27.5	2.1	1		
NO_2 (µg/m^3)	30.2	3.5	13.1	37.0	30.5	28.0	33.0	5.0	0.442	1	
O_3 (µg/m^3)	48.2	4.2	42.8	69.2	46.9	45.3	49.3	4.0	-0.469	-0.758	1
**Non-urban areas (n=371756)**											
PM_{10} (µg/m^3)	22.5	2.9	13.4	28.1	23.6	20.4	24.3	3.9	1		
NO_2 (µg/m^3)	22.4	4.0	8.3	32.7	22.4	19.4	25.6	6.3	0.824	1	
O_3 (µg/m^3)	55.7	4.7	41.9	79.6	55.8	53.7	58.0	4.4	-0.840	-0.866	1

Abbreviations: SD=Standard Deviation; P25 = 25th percentile; P75=75th percentile; IQR=interquartile range.

eTable 4: Association between annual mean levels (2019) of air pollutants and the study endpoints, in the entire sample (left) and after having excluded individuals with exposure attributed at the centroid of their municipality of residency (right).

	All sample (n=709864)			Excluding individuals with geocoding at the centroid of the municipality of residency (sample size: n=697642)		
	**HR[a]**	**95%CI**		**HR[a]**	**95%CI**	
**SARS-CoV-2 infectivity**						
PM_{10}	1.02	1.01	1.03	1.02	1.01	1.03
NO_2	1.02	1.00	1.05	1.02	1.00	1.04
O_3	0.95	0.93	0.97	0.95	0.93	0.97
**COVID-19 Hospitalization**						
PM_{10}	1.00	0.96	1.04	1.00	0.96	1.04
NO_2	1.02	0.95	1.08	1.01	0.95	1.08
O_3	0.96	0.90	1.02	0.96	0.91	1.02
of these: hospitalization requiring ICU/mechanical ventilation						
PM_{10}	0.99	0.94	1.05	0.99	0.94	1.05
NO_2	1.05	0.97	1.14	1.05	0.97	1.14
O_3	0.94	0.87	1.01	0.94	0.87	1.02
**COVID-19 mortality**						
PM_{10}	1.09	1.01	1.17	1.09	1.01	1.18
NO_2	1.18	1.05	1.32	1.20	1.06	1.35
O_3	0.91	0.82	1.02	0.90	0.80	1.00

Abbreviations: ICU=Intensive Care Unit.

[a]: Single-pollutant Cox regression models, adjusting for age, sex, urbanization (urban vs. non-urban), deprivation index (quintiles), positive history of coronary heart disease, stroke, cancer, Chronic Obstructive Pulmonary Disease, treatment for diabetes, anti-hypertensive treatment, treatment for obstructive airway diseases. For the infectivity and the mortality endpoints, age was modelled as a polynomial of degree 3 and 2, respectively.

HR: Hazard ratios for 1 interquartile range (IQR) increase in Province-wide air pollutants. IQR values: PM_{10}=3.5 µg/m^3; NO_2=8.7 µg/m^3; O_3=9.7 µg/m^3

Table e5: Association between long-term exposure to air pollutants and the study endpoints, by pandemic waves, in urban and non-urban areas.

	Urban		Non-urban	
	**First wave**	**Second wave**	**First wave**	**Second wave**
**Infectivity**				
PM_{10}	1.24 (1.10; 1.41)	1.12 (1.08; 1.16)	0.98 (0.91; 1.04)	0.99 (0.98; 1.01)
NO_2	1.29 (1.12; 1.48)	1.02 (0.98; 1.05)	1.02 (0.90; 1.15)	1.02 (0.99; 1.05)
O_3	0.89 (0.78; 1.01)	0.91 (0.88; 0.95)	0.93 (0.83; 1.04)	0.97 (0.94; 1.00)
**Hospitalization**				
PM_{10}	1.56 (1.28; 1.92)	1.11 (1.00; 1.22)	0.94 (0.84; 1.04)	0.97 (0.92; 1.02)
NO_2	1.38 (1.11; 1.71)	1.00 (0.89; 1.11)	0.91 (0.75; 1.10)	1.01 (0.92; 1.11)
O_3	0.79 (0.64; 0.97)	0.93 (0.84; 1.03)	0.97 (0.81; 1.17)	1.00 (0.92; 1.09)
**Mortality**				
PM_{10}	1.56 (1.07; 2.26)	1.16 (0.97; 1.39)	1.04 (0.84; 1.29)	1.06 (0.96; 1.16)
NO_2	1.38 (0.93; 2.06)	1.13 (0.92; 1.38)	1.13 (0.76; 1.68)	1.18 (1.00; 1.41)
O_3	0.72 (0.49; 1.06)	0.92 (0.77; 1.11)	0.86 (0.59; 1.25)	0.94 (0.80; 1.11)

HR= Hazard ratios for 1 interquartile range (IQR) increase in Province-wide air pollutants. IQR values: PM_{10}=3.5 µg/m^3; NO_2=8.7 µg/m^3; O_3=9.7 µg/m^3. HR estimates from single-pollutant time-stratified Cox models including age, sex, urbanization (urban vs. non-urban), deprivation index (quintiles), positive history of coronary heart disease, stroke, cancer, Chronic Obstructive Pulmonary Disease, treatment for diabetes, anti-hypertensive treatment, treatment for obstructive airway diseases, air pollutant and air pollutant*wave interaction. For the infectivity and the mortality endpoints, age was modelled as a polynomial of degree 3 and 2, respectively.
First wave: 01/02-31/05/20. Second wave: 01/06-31/12/20.

eTable 6: Association between annual mean levels (2019) of air pollutants and SARS-CoV-2 infectivity in the entire Province (left) and in urban and non-urban areas (right), in single- and bi-pollutant models.

	**Entire Province[a]**						**Urban areas[b]**						**Non-urban areas[b]**					
	**Single-pollutant**			**Bi-pollutant**			**Single-pollutant**			**Bi-pollutant**			**Single-pollutant**			**Bi-pollutant**		
	**HR**	**95%CI**		**HR**	**95%CI**		**HR**	**95%CI**		**HR**	**95%CI**		**HR**	**95%CI**		**HR**	**95%CI**	
PM_{10}	1.02	1.01	1.03				1.12	1.09	1.16				0.99	0.98	1.01			
Adjusted for NO2				1.02	1.00	1.04				1.17	1.12	1.21				0.95	0.92	0.98
Adjusted for O3				0.97	0.95	0.99				1.16	1.10	1.22				0.93	0.91	0.96
NO_2	1.02	1.00	1.05				1.03	1.00	1.07				1.02	0.99	1.05			
Adjusted for PM10				1.00	0.97	1.04				0.93	0.89	0.97				1.10	1.04	1.16
Adjusted for O3				0.93	0.90	0.97				0.92	0.88	0.97				0.95	0.90	1.01
O_3	0.95	0.93	0.97				0.92	0.89	0.95				0.97	0.94	1.00			
Adjusted for PM10				0.91	0.88	0.95				1.04	0.98	1.10				0.88	0.84	0.92
Adjusted for NO2				0.90	0.87	0.93				0.86	0.82	0.91				0.93	0.88	0.98

[a]: Cox regression models, adjusting for age, sex, urbanization (urban vs. non-urban), deprivation index (quintiles), positive history of coronary heart disease, stroke, cancer, Chronic Obstructive Pulmonary Disease, treatment for diabetes, anti-hypertensive treatment, treatment for obstructive airway diseases. For the infectivity and the mortality endpoints, age was modelled as a polynomial of degree 3 and 2, respectively.
[b]: Stratified Cox regression models in urban and non-urban areas, adjusting for age, sex, deprivation index (quintiles), positive history of coronary heart disease, stroke, cancer, Chronic Obstructive Pulmonary Disease, treatment for diabetes, anti-hypertensive treatment, treatment for obstructive airway diseases. For the infectivity and the mortality endpoints, age was modelled as a polynomial of degree 3 and 2, respectively.
HR: Hazard ratios for 1 interquartile range (IQR) increase in Province-wide air pollutants. IQR values: PM_{10}=3.5 µg/m^3; NO_2=8.7 µg/m^3; O_3=9.7 µg/m^3

eTable 7: Association between annual mean levels (2019) of air pollutants and COVID-19 hospitalizations in the entire Province (left) and in urban and non-urban areas (right), in single- and bi-pollutant models.

	Entire Province[a]						Urban areas[b]						Non-urban areas[b]					
	Single-pollutant			Bi-pollutant			Single-pollutant			Bi-pollutant			Single-pollutant			Bi-pollutant		
	HR	95%CI		HR	95%CI		HR	95%CI		HR	95%CI		HR	95%CI		HR	95%CI	
PM_{10}	1.00	0.96	1.04				1.18	1.08	1.29				0.96	0.91	1.00			
Adjusted for NO2				0.98	0.93	1.01				1.23	1.11	1.37				0.89	0.82	0.97
Adjusted for O3				0.94	0.88	1.00				1.31	1.12	1.52				0.89	0.82	0.96
NO_2	1.02	0.95	1.08				1.06	0.96	1.17				0.99	0.91	1.07			
Adjusted for PM10				1.04	0.94	1.14				0.93	0.83	1.05				1.18	1.02	1.36
Adjusted for O3				0.94	0.85	1.05				0.96	0.83	1.11				0.95	0.81	1.12
O_3	0.96	0.90	1.02				0.91	0.83	1.00				1.00	0.92	1.08			
Adjusted for PM10				0.89	0.81	0.98				1.13	0.96	1.33				0.84	0.74	0.96
Adjusted for NO2				0.92	0.83	1.01				0.87	0.76	1.00				0.95	0.82	1.11

[a]: Cox regression models, adjusting for age, sex, urbanization (urban vs. non-urban), deprivation index (quintiles), positive history of coronary heart disease, stroke, cancer, Chronic Obstructive Pulmonary Disease, treatment for diabetes, anti-hypertensive treatment, treatment for obstructive airway diseases. For the infectivity and the mortality endpoints, age was modelled as a polynomial of degree 3 and 2, respectively.
[b]: Stratified Cox regression models in urban and non-urban areas, adjusting for age, sex, deprivation index (quintiles), positive history of coronary heart disease, stroke, cancer, Chronic Obstructive Pulmonary Disease, treatment for diabetes, anti-hypertensive treatment, treatment for obstructive airway diseases. For the infectivity and the mortality endpoints, age was modelled as a polynomial of degree 3 and 2, respectively.
HR: Hazard ratios for 1 interquartile range (IQR) increase in Province-wide air pollutants. IQR values: PM_{10}=3.5 µg/m^3; NO_2=8.7 µg/m^3; O_3=9.7 µg/m^3

eTable 8: Association between annual mean levels (2019) of air pollutants and COVID-19 mortality in the entire Province (left) and in urban and non-urban areas (right), in single- and bi-pollutant models.

	**Entire Province[a]**						**Urban areas[b]**						**Non-urban areas[b]**					
	**Single-pollutant**			**Bi-pollutant**			**Single-pollutant**			**Bi-pollutant**			**Single-pollutant**			**Bi-pollutant**		
	**HR**	**95%CI**		**HR**	**95%CI**		**HR**	**95%CI**		**HR**	**95%CI**		**HR**	**95%CI**		**HR**	**95%CI**	
PM_{10}	1.09	1.01	1.17				1.22	1.04	1.42				1.05	0.96	1.14			
Adjusted for NO2				1.01	0.91	1.13				1.20	0.99	1.46				0.94	0.81	1.10
Adjusted for O3				1.09	0.96	1.24				1.41	1.06	1.86				1.05	0.91	1.22
NO_2	1.18	1.05	1.32				1.18	0.99	1.41				1.17	1.00	1.37			
Adjusted for PM10				1.16	0.98	1.37				1.05	0.84	1.31				1.282	0.98	1.69
Adjusted for O3				1.25	1.03	1.51				1.15	0.89	1.48				1.398	1.04	1.88
O_3	0.91	0.82	1.02				0.89	0.76	1.05				0.93	0.80	1.08			
Adjusted for PM10				1.01	0.84	1.21				1.19	0.88	1.61				0.99	0.77	1.28
Adjusted for NO2				1.08	0.90	1.29				0.97	0.77	1.23				1.22	0.92	1.61

[a]: Cox regression models, adjusting for age, sex, urbanization (urban vs. non-urban), deprivation index (quintiles), positive history of coronary heart disease, stroke, cancer, Chronic Obstructive Pulmonary Disease, treatment for diabetes, anti-hypertensive treatment, treatment for obstructive airway diseases. For the infectivity and the mortality endpoints, age was modelled as a polynomial of degree 3 and 2, respectively.

[b]: Stratified Cox regression models in urban and non-urban areas, adjusting for age, sex, deprivation index (quintiles), positive history of coronary heart disease, stroke, cancer, Chronic Obstructive Pulmonary Disease, treatment for diabetes, anti-hypertensive treatment, treatment for obstructive airway diseases. For the infectivity and the mortality endpoints, age was modelled as a polynomial of degree 3 and 2, respectively.

HR: Hazard ratios for 1 interquartile range (IQR) increase in Province-wide air pollutants. IQR values: PM_{10}=3.5 µg/m^3; NO_2=8.7 µg/m^3; O_3=9.7 µg/m^3

eFigure 1: DAG for the assessment of the association between long-term air pollution (2019 annual mean) and SARS-CoV-2 infectivity (positive test). DAG drawn using DAGitty.
Panel A (above): Model 1, potential confounders; panel B (below): Model 2, potential confounders and mediators.

A: Model 1: potential confounders

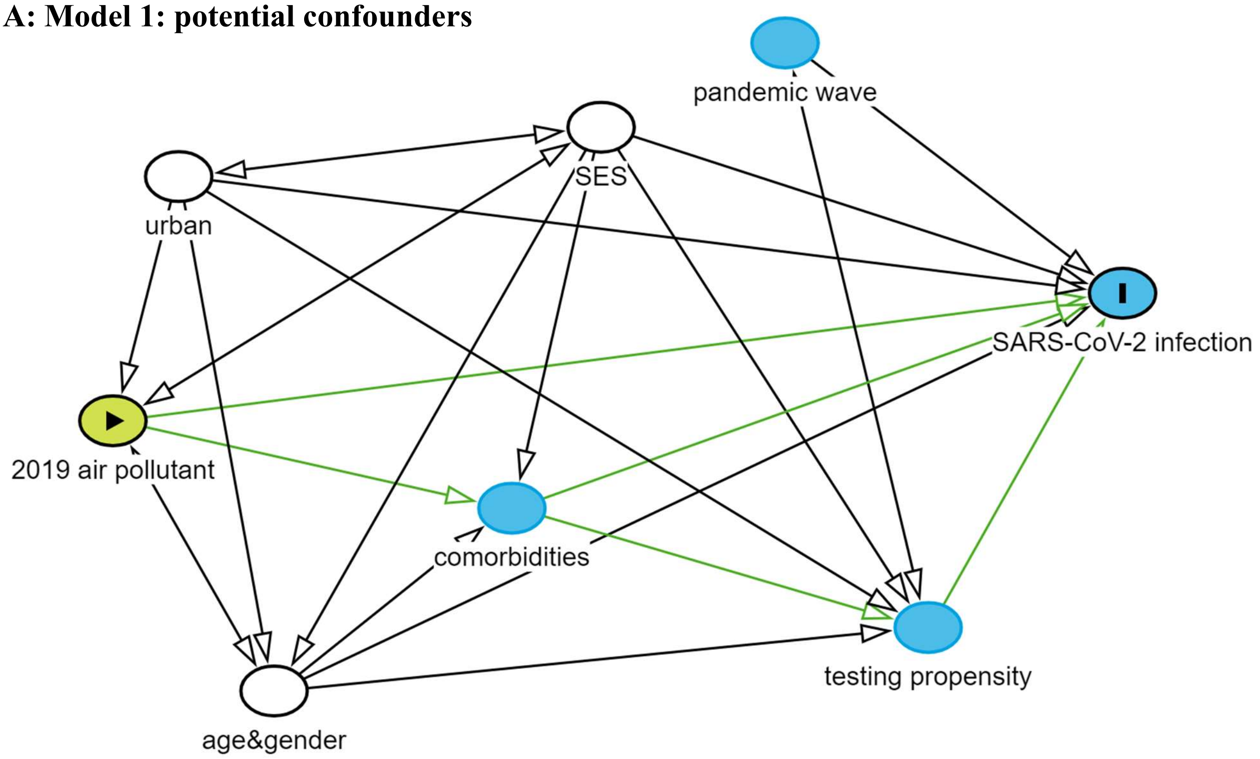

B. Model 2: potential confounders and mediators

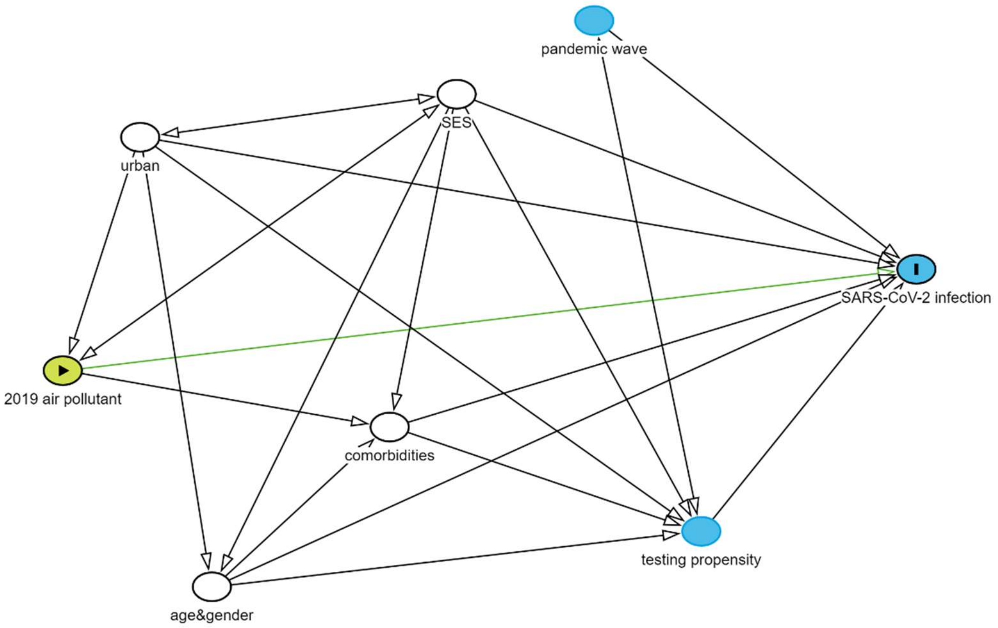

All the results in the main text and in the supplementary tables refer to Model 2.

eFigure 2: Temporal trend in the weekly number of cases in the Varese Province and in the study population, from 01/02 to 31/12/2020.

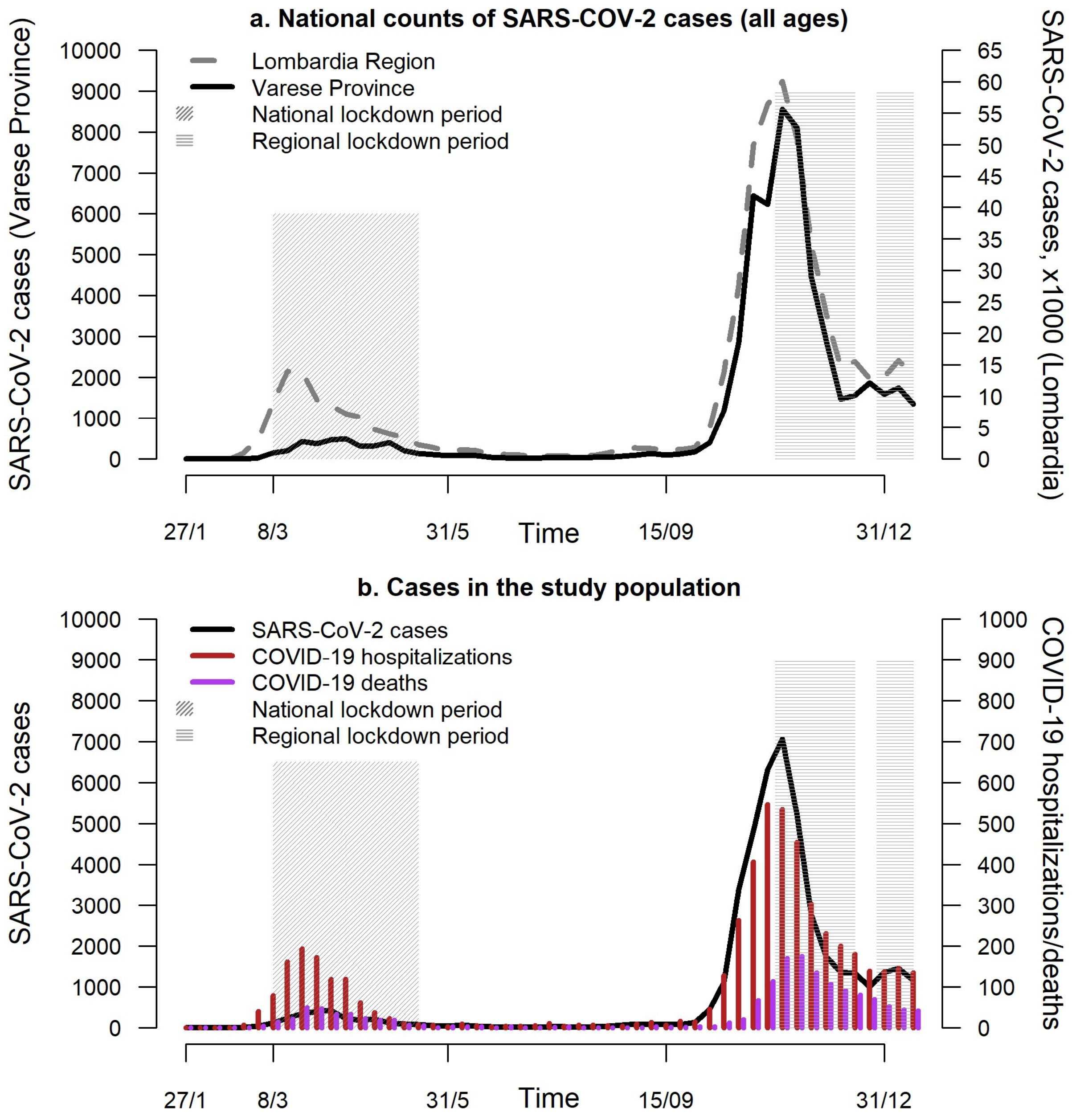

(A) Weekly number of SARS-CoV-2 cases in the Province of Varese (left vertical axis) and in the Lombardy region (right vertical axis), according to the official statistics, including all ages; and (B) weekly number of SARS-CoV-2 cases (left vertical axis), COVID-19 hospitalizations and deaths (rigth vertical axis) in the study population (18+ years old, excluding subjects living in a residental care home). National lockdown and regional mobility restriction periods are indicated as shaded areas. The national lockdown period lasted from 8 March to 17 May 2020. There were no mobility restrictions from May to October 2020. Thereafter, intermittent periods at different levels of mobility restrictions were in place from November 2020 to March 2021 in the Lombardy region, but these were not as stringent as during the national lockdown

eFigure 3: Spatial distribution of the 2019 annual mean levels of PM_{10} in the Varese Province area, colored by sample deciles.

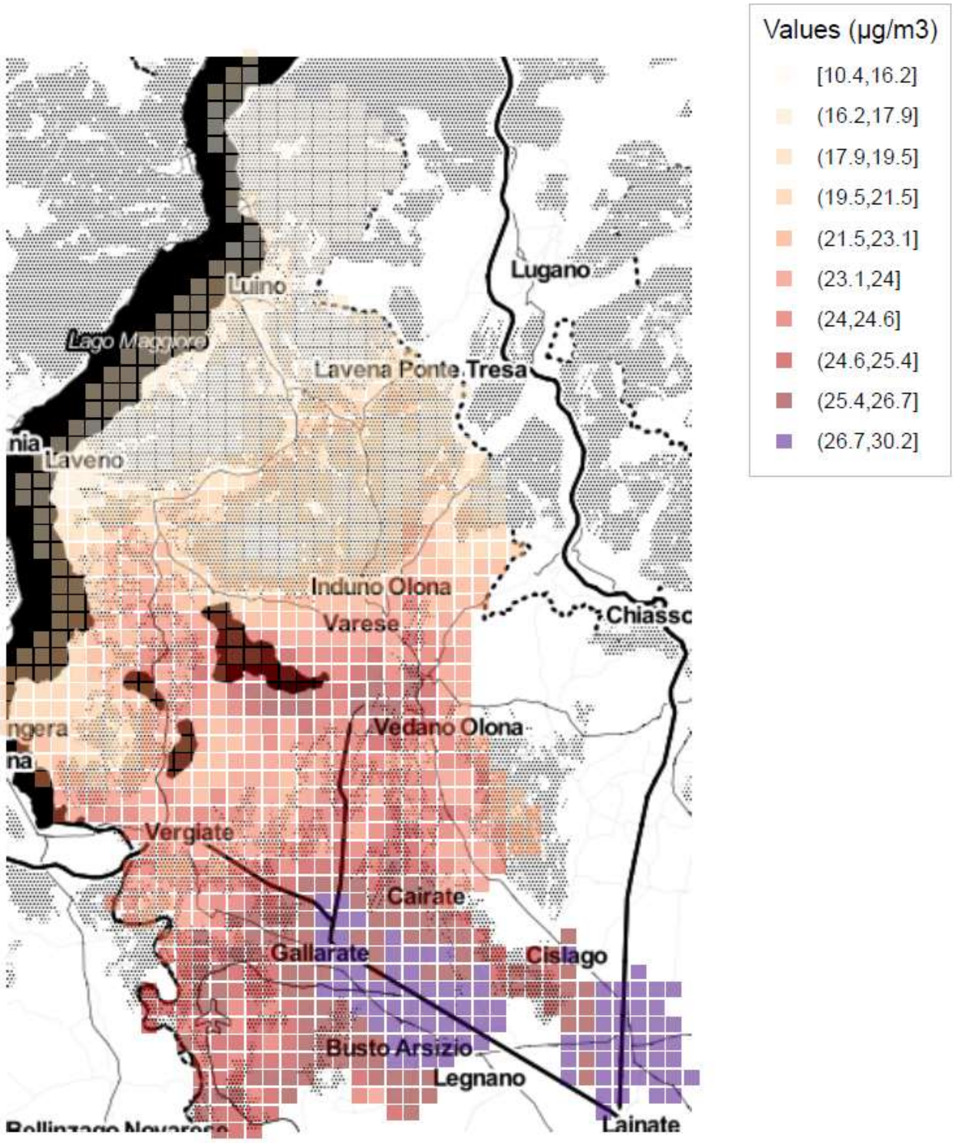

eFigure 4: Spatial distribution of the 2019 annual mean levels of NO_2 in the Varese Province area, colored by sample deciles.

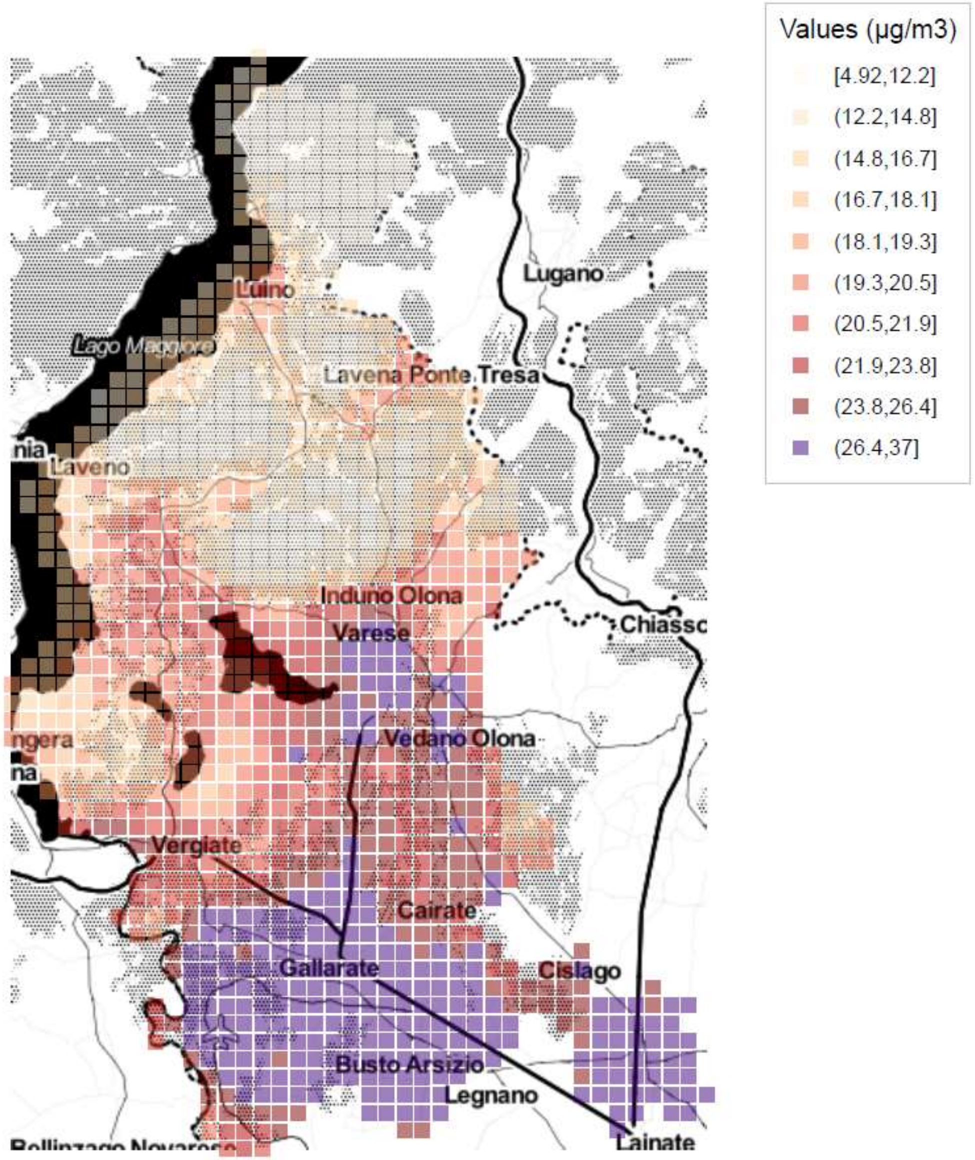

eFigure 5: Spatial distribution of the 2019 annual mean levels of O_3 in the Varese Province area, colored by sample deciles.

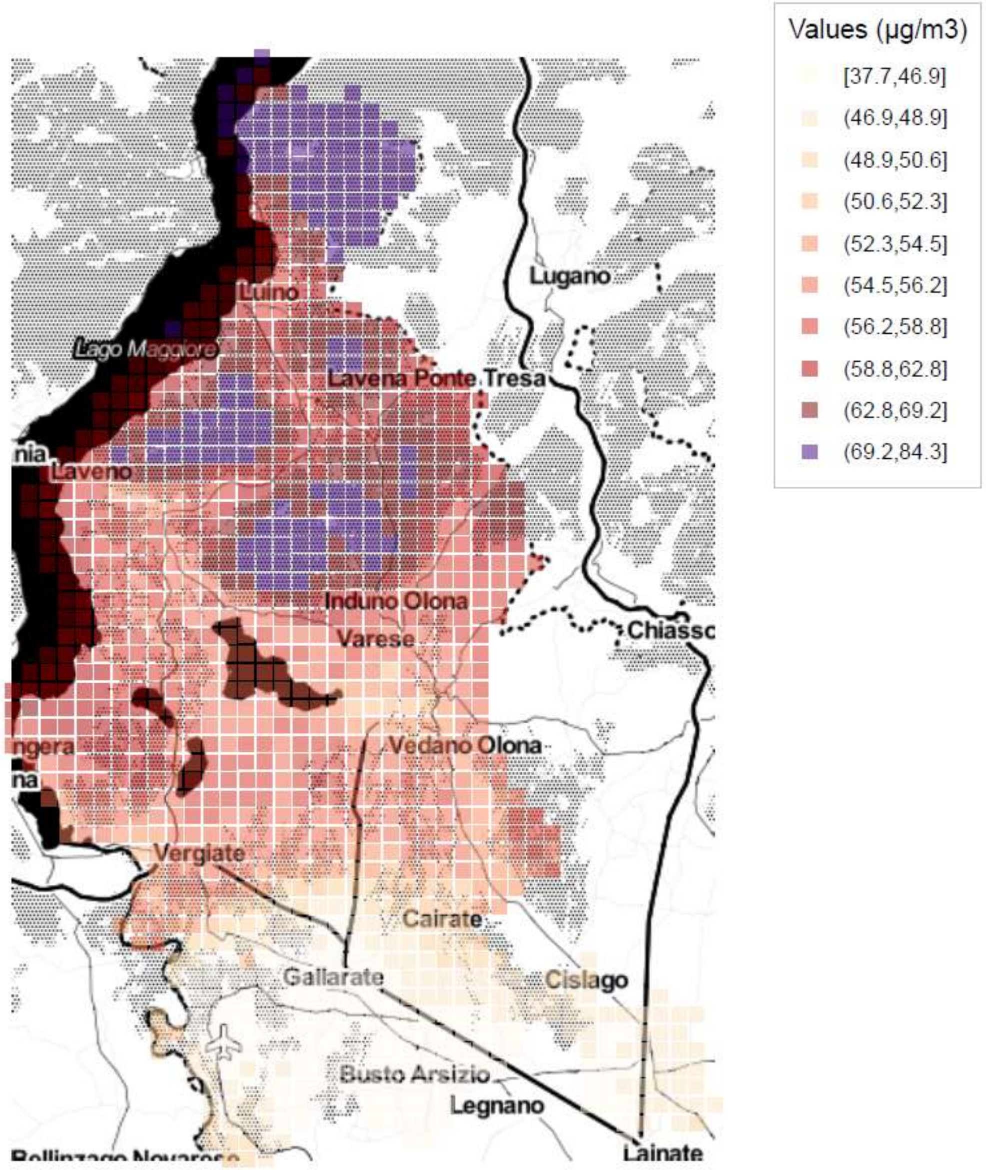

eFigure 6: Modeling of COVID-19 hospitalization risk due to 1μg/m[3] increase in one pollutant by urban and non-urban specific quintiles of the co-pollutant.

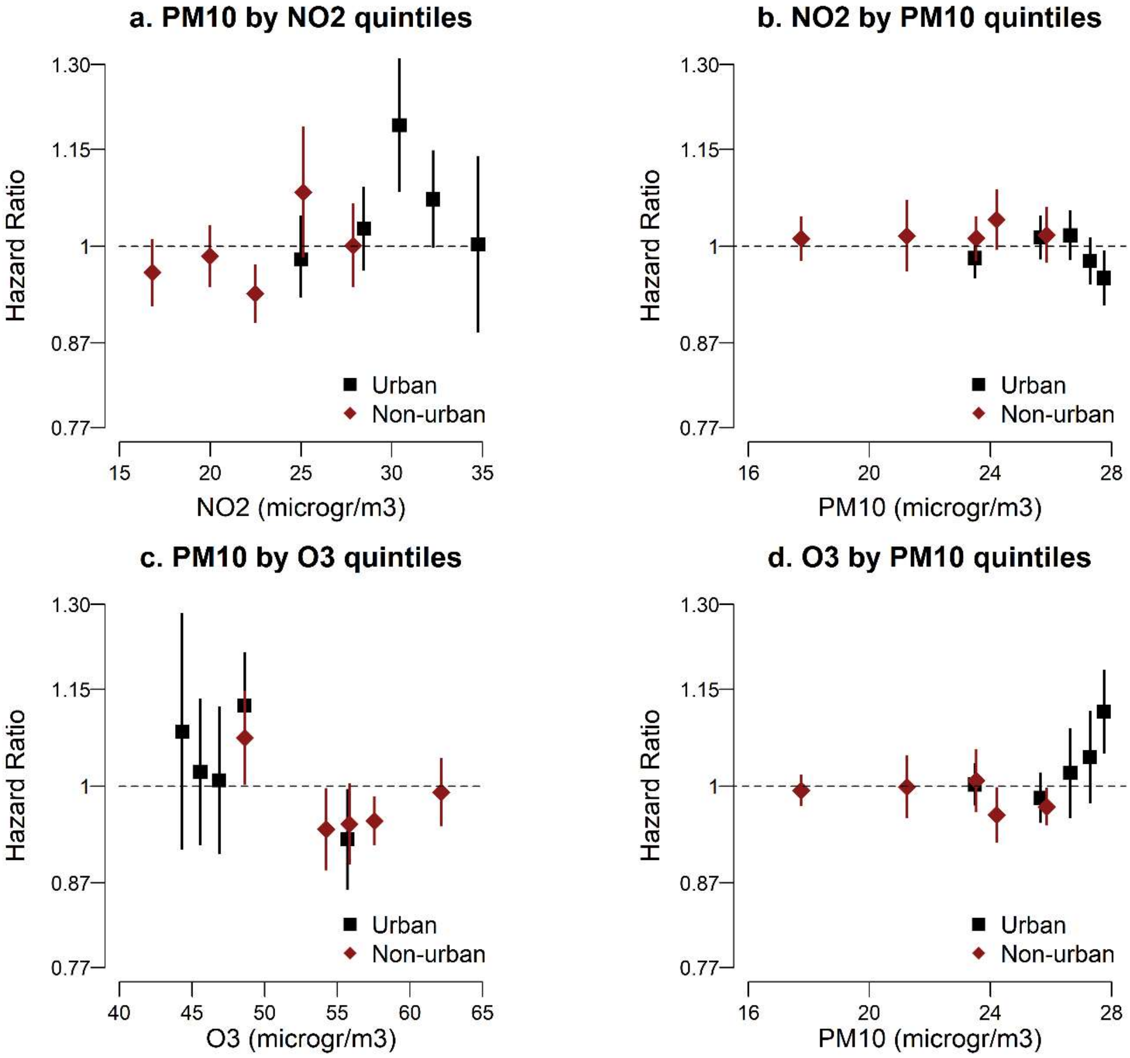

eFigure 7: Modeling of COVID-19 mortality risk due to 1 μg/m^3 increase in one pollutant by urban and non-urban specific quintiles of the co-pollutant.

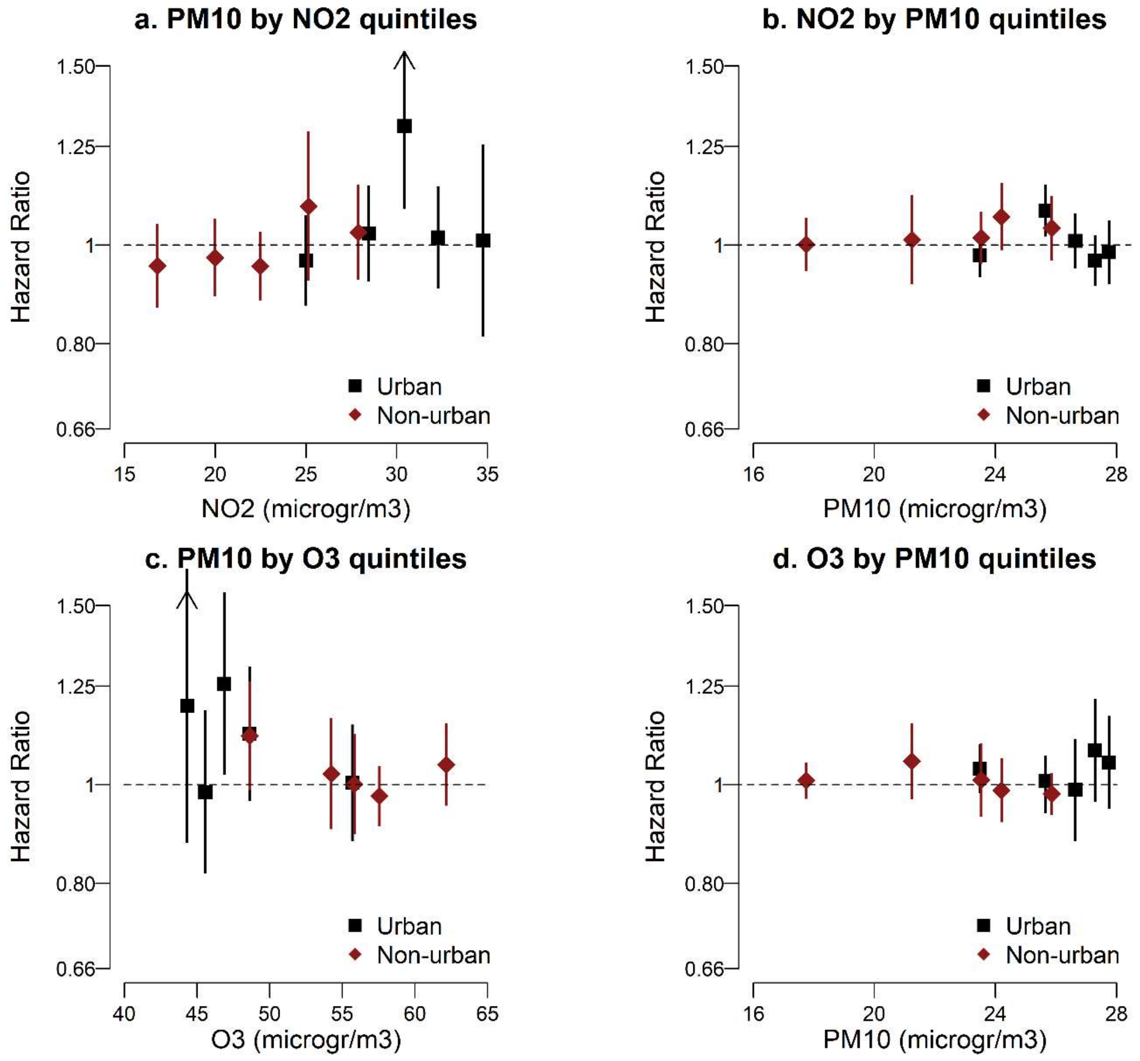

Supplement: Supplementary file 2 [file ede-36-11-s002.pdf]
